# High-light gaps and carbon allocation regulate regeneration of understory *Fraxinus mandshurica* seedlings

**DOI:** 10.3389/fpls.2026.1936089

**Published:** 2026-09-03

**Authors:** Shixiong Wu, Fangfei Wu, Chunchao Dong, Zhihong Gao, Lulu He, Qijing Liu

**Affiliations:** 1 College of Landscape Architecture, Changchun University, Changchun, China; 2 College of Forestry, Beijing Forestry University, Beijing, China

**Keywords:** biomass allocation, carbon gain, nonstructural carbohydrates, seedling regeneration, survival strategy

## Abstract

Against the backdrop of climate warming, understanding the regeneration mechanisms of seedlings under forest canopies is crucial for the sustainable development of temperate forests. Broad-leaved Korean pine forests serve as the zonally climax vegetation in the Changbai Mountains, however, the causes of regeneration barriers for their primary tree species remain unclear. In particular, while shade-tolerant *Fraxinus mandshurica* seedlings struggle to regenerate in primary forests, they regenerate well in secondary poplar-birch forests. Nevertheless, the underlying mechanism responsible for this phenomenon remains unclear. This study employed a combined approach of understory light environment monitoring and controlled nursery experiments to observe seedling survival rates, growth characteristics, seasonal dynamics of nonstructural carbohydrate (NSC)., and carbon assimilation over two consecutive years. The results indicated that *F. mandshurica* seedlings in secondary forests adopt a strategy of investing more in specific leaf area and above ground biomass during summer, allowing them to survive longer during the canopy closure period. High light availability in early spring and late autumn and the concomitant positive carbon gain drive carbon accumulation in secondary forest seedlings, with primary forest seedlings attaining lower annual carbon gain. Throughout the growing season, NSC concentration in seedlings showed a trend of decreasing in summer and increasing in autumn, with most NSC being stored in the root system before dormancy. Nursery control experiments confirmed that simulating the light environment of secondary forests can improve seedling growth, survival rate, and NSC pools. In summary, this study demonstrates that *F. mandshurica* seedlings in secondary forests realize net carbon accumulation through phenological escape under high light in early spring and late autumn. During summer canopy closure, they maintain positive carbon gain by increasing aboveground biomass allocation and specific leaf area, and accumulate abundant NSC in perennial branches in autumn for overwintering.

## Introduction

Nonstructural carbohydrates (NSCs) are the primary products of photosynthesis and are stored in various organs as starch and soluble sugars (Xu et al., 2022; Dalgleish et al., 2023; Zhou et al., 2023). Studies have shown that seedlings with higher NSC storage capacity are more likely to survive under low light or canopy disturbance, reflecting a trade-off between growth rate and low-light tolerance (Tripathi et al., 2020). NSCs exhibit distinct seasonal dynamics driven by seasonal variations in resources such as light, temperature, and water (Zhang et al., 2020). In the early growing season, seedlings prioritize the allocation of stored NSCs to the formation of new leaves and shoots to meet the carbon demands of leaf expansion and early growth (Puglielli et al., 2021). Upon entering the summer period of dense canopy closure, light becomes the primary limiting factor, and the seedlings’ photosynthetic carbon assimilation capacity declines significantly (Zhou et al., 2023). At this stage, they rely primarily on NSC reserves accumulated in spring to sustain basal respiratory metabolism (Schuster et al., 2021; Lee et al., 2022). Whereas in autumn, as the canopy reopens and light conditions improve, the seedlings resume efficient photosynthesis, prioritizing the storage of synthesized NSC in the root system and perennial organs to reserve carbon sources for overwintering dormancy and leaf unfurling in the following spring. Previous studies have predominantly employed pot experiments, where restricted root growth may lead to underestimation of root starch concentrations (Zhou et al., 2020; Barrere et al., 2021). Therefore, the *in situ* experimental approach adopted in this study is crucial for fully elucidating the growth patterns of seedlings under forest canopies (Ji et al., 2021).

Light is a critical factor for the natural regeneration of understory seedlings, it not only directly influences seedling survival and establishment but also significantly shapes the heterogeneity of the forest environment (Amico Roxas et al., 2021; Lee and Ibáñez, 2021). Light exerts this influence primarily by regulating seedling growth and biomass allocation through the light transmittance of the forest canopy. Whether this effect is promoting or inhibitory depends on the species’ inherent shade tolerance and light requirements (Yin et al., 2023). It is well known that temperate deciduous forests have a distinctive feature, light levels beneath the canopy are not static but exhibit marked seasonal fluctuations (Fernández-Milmanda and Ballaré, 2021; Luo et al., 2022; Fan et al., 2024). Specifically, in spring, before the canopy has fully unfurled its leaves, a large amount of direct sunlight can penetrate the canopy to reach the forest floor understory plants utilize phenological escape strategies to exploit these brief high-light gaps. Studies indicated that numerous understory species, including both spring ephemeral herbs and tree seedlings, rely on these gaps to acquire a substantial portion often 50% to 100% of their annual carbon budget before canopy closure (Zhang et al., 2022). By summer, the canopy closes completely, and understory light drops to its annual minimum, typically accounting for less than 2% of annual light. By autumn, as leaves gradually fall, the canopy reopens, and another autumn high-light gap emerges (Liu et al., 2023). Notably, autumn phenological escape is equally important some species maintain photosynthetic activity even after the canopy reopens, using this gap to stockpile carbon reserves for overwintering (Umaña et al., 2021). These phenologically driven seasonal changes in light have a profound impact on the survival strategies of understory seedlings. Research indicates that shade-tolerant tree species perform better under low-light conditions largely because they can utilize limited light resources more efficiently, such as by capturing light energy through larger individual leaf areas. This reflects a classic ecological trade-off: shade-tolerant species grow faster than shade-intolerant species under low light, and vice versa a phenomenon long considered the core explanation of shade tolerance theory (Yin et al., 2023). However, some scholars have proposed a different perspective: in low-light environments, survival may be more important than growth (Durand et al., 2024). Shade-tolerant species may not necessarily grow faster under low light, but they often have higher survival rates, primarily due to their efficient carbon allocation and storage capabilities (Sangsupan et al., 2021). During the high-light gap in spring, seedlings seize the opportunity to rapidly perform photosynthesis and accumulate carbon reserves, as the summer canopy closure period begins and light becomes severely limited (Vitasse et al., 2021; Fan et al., 2024). Seedlings rely on carbohydrates stored in spring to maintain their basal metabolism. When the high-light gap returns in autumn, seedlings resume efficient photosynthesis and prioritize storing synthesized nutrients in their root systems to prepare for overwintering and leaf unfurling the following year. This seasonal carbon dynamic, characterized by accumulation in spring, consumption in summer, and storage in autumn, plays a decisive role in the survival of understory seedlings. It ultimately determines whether seedlings can successfully regenerate in low-light environments (Umaña et al., 2021).

Broad-leaved Korean pine forests are renowned for having the richest tree species diversity and the most stable stand structure in the northern temperate zone. However, difficulties in the regeneration of dominant tree species have long been a major challenge (Wang and Wang, 2023). *Fraxinus mandshurica* is a dominant species in broad-leaved Korean pine forests and an important timber species in Northeast China (Liu et al., 2020a; Schuster et al., 2021). Studies indicate that while seed density and seedling density are positively correlated for this species, the density of one-year-old seedlings gradually decreases due to intraspecific competition (Long et al., 2021; Dalgleish et al., 2023; Peng et al., 2024). Furthermore, a negative density constraint effect resulting from the combined influence of density limitation and habitat filtering is prevalent in both forest stands (Fan et al., 2024). *F. mandshurica* grows well under ample light but faces impeded regeneration in shaded environments, therefore, elucidating its regeneration mechanisms is of great significance (Wang and Wang, 2023; Li et al., 2024). Previous studies have provided some clues, for example, secondary poplar-birch forests serve as ideal sites for *F. mandshurica* seedling regeneration due to the abundant light resulting from the deciduous nature of poplars and birches, as well as the enhanced scattered light reflected by birch bark. Long-term monitoring also indicates that while *F. mandshurica* has virtually no regeneration layer in primary forests, it regenerates well in secondary forests. However, puzzlingly, PAR data show that light conditions in secondary forests are not significantly better than those in primary forests so how exactly do *F. mandshurica* seedlings regenerate in relatively shaded understory environments? To address this question, we conducted multi-year *in situ* monitoring experiments in the understory, This seasonal carbon dynamic, characterized by accumulation in spring, consumption in summer, we systematically assessed seasonal variations in NSCs and biomass allocation trade-offs in seedlings. Based on these findings, we aim to answer the following questions: (i) How do the spring and autumn high-light gaps in temperate deciduous forests drive the phenological escape strategy of *F. mandshurica* seedlings, and how do these phenological differences ultimately determine seedling survival and regeneration. (ii) How do the contrasting biomass allocation strategies between secondary and primary forests affect the survival of *F. mandshurica* seedlings under low-light conditions, and why does greater aboveground allocation confer an advantage for survival under shaded conditions. (iii) How do *F. mandshurica* seedlings accumulate carbon reserves in spring and utilize stored NSC to sustain respiratory consumption during the summer canopy closure period, and why does enhance autumn light availability increase NSC storage and seedling survival. To test these hypotheses, this study integrates *in situ* light measurements with leaf carbon gain simulations, aiming to provide a novel quantitative description of the photosynthetic niche of *F. mandshurica*.

## Materials and methods

### Study site and species

The study area is in the Changbai Mountain National Nature Reserve in Jilin Province. The study site lies at an altitude of 750 meters above sea level, with a climate featuring cold and windy winters coupled with humid summers. Most of the rain falls between June and September. The average temperature is around 13.7 °C. April is usually cold while May to October is warm and July is the hot month. The main area of study is the broad-leaved Korean pine forest zone. The soil here is mountainous dark brown forest soil. *F. mandshurica* is a dominant species in broad-leaved Korean pine forests. Its seedlings regenerate poorly under the primary forest canopies but grow well in the secondary poplar-birch forests, which is why it was selected for this study. *F. mandshurica* trees are special because they unfold their leaves early and shed them late. Based on observations showed that leaf expansion initiated on May 12 and concluded on May 30, with leaf senescence occurring on September 17. In addition, forest canopy went through three successive phenological stages: early spring leaf unfolding (May 7 to May 25), full canopy closure from May 26 to September 5, and progressive leaf abscission starting on September 6.

### Understory plant recruitment survey

The study site is located in a poplar-birch forest and a primary broad-leaved Korean pine forest on the northern slope of the Changbai Mountain Nature Reserve. The forests are around 90 and 300 years old, respectively. A one-hectare permanent plot was set up in the forest in May 2010 and visited again in 2020. We established a permanent plot of the same size in the secondary forest for comparative purposes in 2021. During the plot survey, we divided each plot into 10m × 10m subplots and conducted a detailed inventory of all trees (DBH ≥5 cm) within each subplot. The coordinates, species names and diameters of the trees were recorded. In July 2021, a survey of *F. mandshurica* seedlings was conducted across 200–1 m × 1m subplots spaced 5m apart.

### Microclimate monitoring

Microclimatic variables including photosynthetic photon flux density, air temperature, soil temperature and soil relative humidity were automatically monitored from 2021 to 2022, with three sensors deployed per plot for each parameter. HOBO Micro Station Loggers (H21-002, Onset Computer Corporation, USA) equipped with PAR sensors (S-LIA-M003) and temperature probes (S-TEB-M002) were mounted 30cm above the ground in the both primary and secondary forest. Continuous readings were aggregated into 30-minute averages. For soil temperature and soil relative humidity (sensors buried 10cm underground), 30-minute data from three replicated plots were further averaged to obtain daily mean values.

### Growth dynamics of nonstructural carbohydrates

#### Growth performance

From June to October 2021, *F. mandshurica* seedlings were collected from the broad-leaved Korean pine forests and secondary poplar-birch forests. 20 seedling samples were collected monthly. The height and ground-level diameter and leaf area of the seedlings were recorded. The seedlings were then taken apart into leaves, current-year and annual and perennial branches and roots. The specific leaf area was also calculated. All parts of the seedlings were dried, weighed, and ground into powder for later use. In May 2021, 200F*. mandshurica* seedlings were marked in each of the two forests. The survival rates of the seedlings were recorded during the spring, summer and fall seasons of 2021-2022.

#### Nonstructural carbohydrates

Soluble sugars and starch were quantified using the anthrone-sulfuric acid method. Powder samples (0.1g) from each organ were placed in 15 mL centrifuge tubes and extracted with 5 mL of 80% ethanol in a water bath at 80°C for 30min. After cooling, the mixture was centrifuged at 4000 rpm for 10min, and the supernatant was collected. The residue was re-extracted twice with 80% ethanol, and the combined supernatants were made up to 50 mL. A 10 mL aliquot was taken for soluble sugar determination. The residue was dried, then mixed with 3 mL of distilled water and gelatinized in a water bath at 100°C for 15min. After adding 2 mL of 9.2 mol·L^−1^ HClO_4_ and shaking for 15min, 4 mL of distilled water was added and the mixture was centrifuged for 10min; the supernatant was collected. The residue was extracted once more with 2 mL of 4.6 mol·L^−1^ HClO_4_, and the combined supernatants were made up to 50 mL. The concentration was determined colorimetrically at 620 nm using the anthrone method against a glucose standard curve. The NSC pool of each organ was calculated as the product of organ biomass and NSC concentration.

### Photosynthesis measurement

#### Photosynthesis

To study the photosynthetic capacity of the seedlings, three healthy *F. mandshurica* seedlings were selected from each of the primary and secondary forests, respectively. The light response curves of the seedlings were measured twice a month during the 2021 and 2022 growing seasons. The measurements were taken on mornings using a portable photosynthetic measurement system. The leaves were induced with light for 15 minutes before measurement. The leaf chamber was maintained at a CO_2_ concentration of 400 μmol·CO_2_·mol^−1^ and an airflow rate of 500 μmol·s^−1^. The leaf temperature was maintained at the level as the ambient temperature.

The light response curve was fitted using a non-rectangular hyperbolic model, as shown below:

(2-1) $$A\left(I\right)=\left(\alpha I+A\max-{\left({\left(\alpha I+A\max\right)}^{2}-4\theta \alpha \mathrm{IA}\max\right)}^{1/2}\right)/\left(2\theta –\mathrm{Rd}\right)$$

Where *A (I)* is the net photosynthetic rate (μmol·m^−2^·s^−1^), *I* is the PAR (μmol·m^−2^·s^−1^), *θ* is the inflection angle of the curve*, α* is the apparent quantum efficiency, *A_max_* is the maximum net photosynthetic rate, and *R_d_* is the dark respiration rate (μmol·m^−2^·s^−1^).

### Carbon assimilation simulation

To study the carbon assimilation of the seedlings the daily carbon assimilation was calculated using the light-temperature coupling model. The model used data on light and temperature recorded throughout the growing season. The calculation formula was used to estimate the carbon assimilation of individual seedlings.

The calculation formula is as follows:

(2-2) $$\text{Daily carbon assimilation }\left(\text{g }{\text{m}}^{-2}\right)\text{ = }\sum_{i}^{1-48}A{\left(I\text{, }T\right)}_{\text{i}}\times \;1800\;\times \;\left(12\times 10^{-6}\right)\;$$

(2-3) $$\text{Daily carbon assimilation}\left({\text{g plant}}^{-1}\right)=\text{Daily carbon assimilation}\left({\text{g m}}^{-2}\right)\times \text{LA}$$

Where *I* and *T* represent the PAR and temperature recorded at 30-minute intervals, respectively. *i* denotes the number of 30-minute intervals in a day (1–48). Constant terms: 1800 represents the conversion from 30 minutes to seconds (s), 12×10^−6^ represents the conversion from mmol to grams (g), *LA* represents the monthly leaf area of the species (calculated during the leaf-expansion period using a leaf area model).

### Nursery simulation experiment

To study the effects of light patterns on the growth and survival of *F. mandshurica* seedlings, a nursery simulation experiment was conducted. One-year-old seedlings were transplanted to the site in early May 2021. Four light treatments were established, simulated secondary forest, simulated primary forest, full sunlight (bare ground), and simulated primary forest with enhanced autumn light. Each treatment had three replicates, with 100 seedlings per replicate. Potting soil was identical to forest soil, and *F. mandshurica* seedlings were watered, fertilized, and protected from weeds and pests during the growing season. The shading control experiment began simulating the autumn light enhancement experiment in September 2021. The growing season was divided into three phases based on canopy phenology, spring (May 4 to May 25), summer (May 25 to September 15), and autumn (September 15 to October 10). During the experiment, shade nets with three needle, four needle, and double four needle configurations were used to represent light transmittance of 40%, 20%, and 4%, respectively, with 100% indicating full illumination. The actual photosynthetic photon flux density (PPFD) under each shade net and in full sunlight was measured using PAR sensors to calculate the light transmittance. Destructive sampling was carried out in each plot on three dates: spring (May 1), summer (August 10) and autumn (October 12). At each sampling time point, 10F*. mandshurica* seedlings per treatment were harvested for NSC analysis. Leaf area was scanned and quantified using a flatbed scanner in summer. The remaining seedlings were retained to monitor their growth performance and survival rate. Harvested seedlings were dissected into leaves, current-year and one-year-old twigs, coarse roots and fine roots.

### Data analysis

Linear mixed models (LMMs) were constructed to assess the independent and interactive effects of forest type, growing stage on seedling growth traits, including height, basal diameter, leaf area, specific leaf area, total biomass, root-shoot ratio, biomass allocation across organs, as well as the pool and concentration of NSCs. LMM analysis was implemented via the lme4 package in R. Seedling ID was set as a random factor in the models to eliminate the confounding influence of individual seedling variation on seasonal growth. One-way ANOVA was adopted to test the effects of treatments under different forest types (2021 and 2022) on seasonal carbon acquisition of seedlings, followed by Fisher’s LSD *post-hoc* test for multiple comparisons among treatment groups. Prior to statistical testing, normality and homogeneity of variance were verified for all datasets. Logarithmic or square-root transformation was conducted on non-conforming data to satisfy the prerequisites of parametric tests. Differences with *P*<0.05 were regarded as statistically significant. All statistical analyses were completed in R 4.2.3 (R Core Team, 2023), and all figures were plotted using Origin 2021.

## Results

### Seasonal light intensity

**Figure 1:**
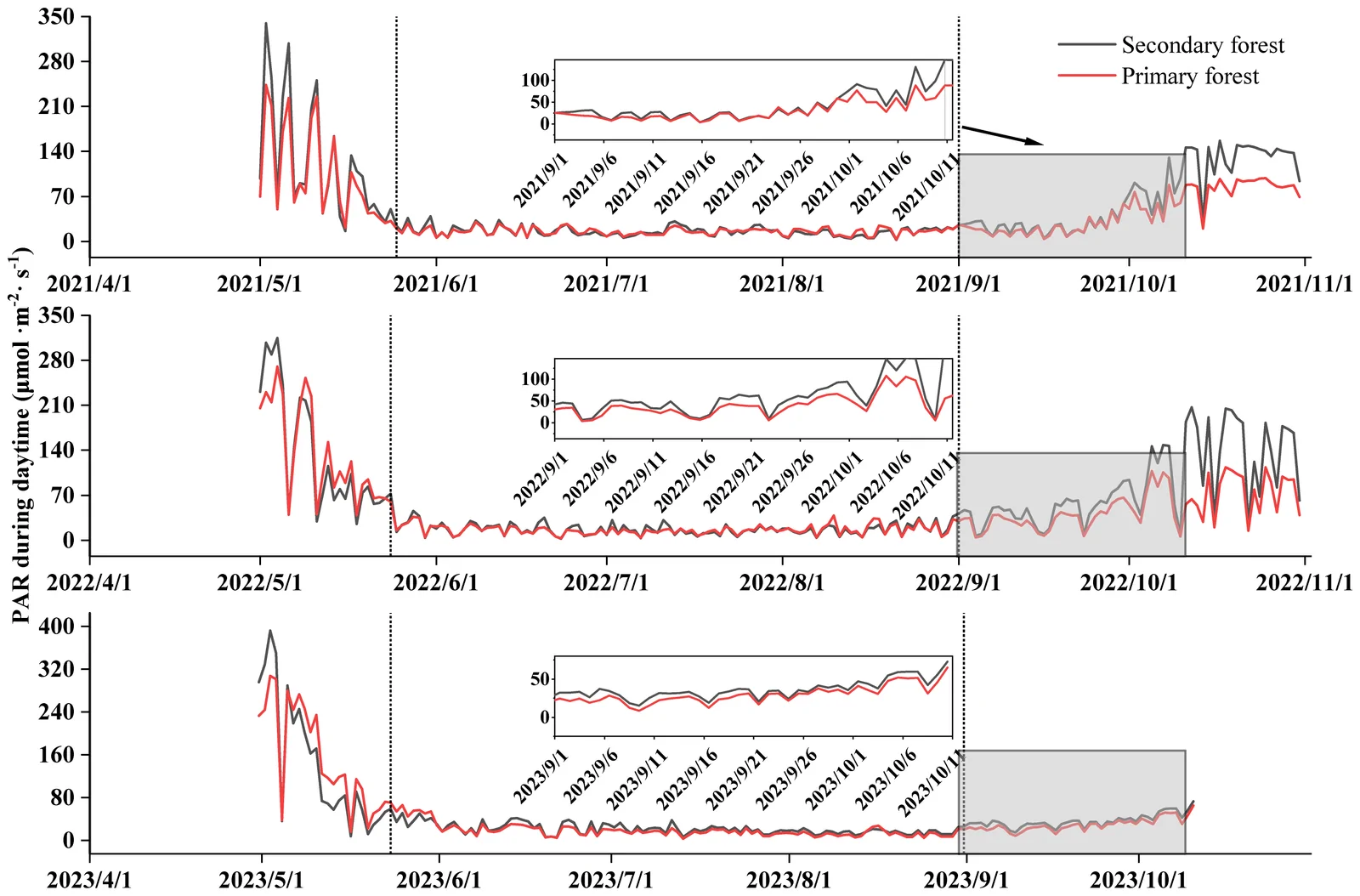
Seasonal variations in daytime (5:30 a. m. to 5:30 p. m.) understory PAR in secondary and primary forests during the growing seasons of 2021 (top), 2022 (middle), and 2023 (bottom). The dotted line on the left and the dotted line on the right indicate the beginning of canopy closure (May 25) and the onset of senescence in the secondary forest canopy (September 1), respectively. The local magnification refers to the period from September 1 to October 11.

Three years of PAR monitoring data under the forest canopy indicate that light availability was higher in both forest types during spring and autumn than in summer. In the secondary forest in 2021, light availability ranged from May 7 to May 25 (87. 26 ± 14. 15 μmol·m^−2^·s^−1^) and in autumn from September 1 to October 7 (40. 46 ± 5. 13 μmol·m^−2^·s^−1^) were significantly higher than those in the primary forest in spring (78. 60 ± 12. 90 μmol·m^−2^·s^−1^) and autumn (30. 559 ± 3. 39 μmol·m^−2^·s^−1^) in the primary forest. In 2022, the autumn canopy light availability in the secondary forest (58. 92 ± 6. 34 μmol·m^−2^·s^−1^) was significantly higher than that of the primary forest in autumn (39. 72 ± 3. 98 μmol·m^−2^·s^−1^). In 2023, the autumn light availability under the canopy of the secondary forest (34. 10 ± 1. 66 μmol·m^−2^·s^−1^) was slightly higher than that of the primary forest in autumn (27. 54 ± 1. 65 μmol·m^−2^·s^−1^) (**Figure 1**).

### Seedling distribution

**Figure 2:**
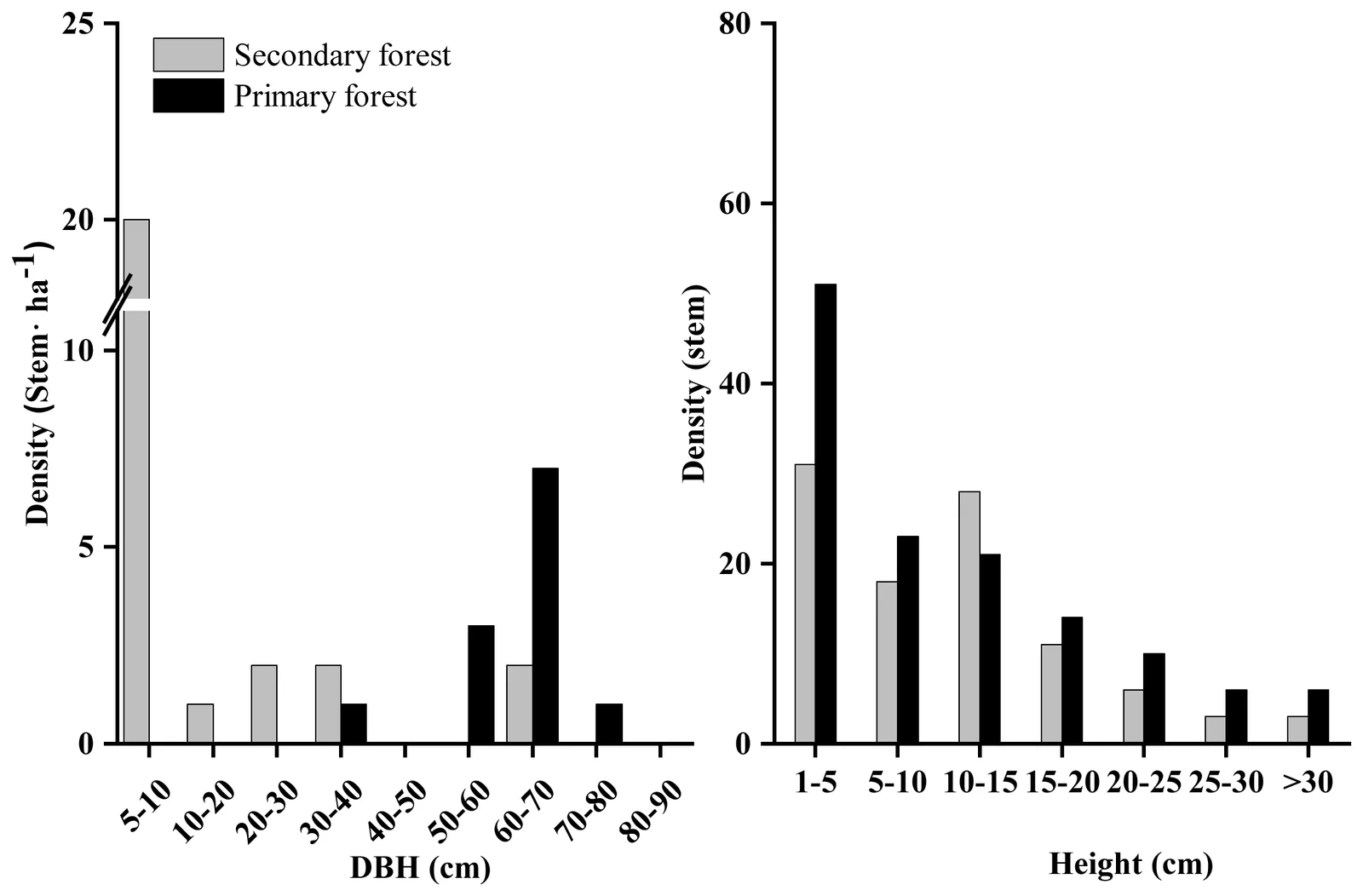
Distribution of DBH and seedling height distribution of *F. mandshurica* in secondary and primary forests.

The basal area of the primary forest (43. 5 m^2^·ha^−1^) was significantly greater than that of the secondary forest (33. 7 m^2^·ha^−1^), whereas the tree density of the secondary forest (1, 159 trees·ha^−1^) was significantly higher than that of the primary forest (587 trees·ha^−1^) (**Table 1**). Total soil carbon and total soil nitrogen in the primary forest were significantly higher than those in the secondary forest, two years of PAR data indicate that light conditions under the canopy of the primary forest during the growing season were inferior to those of the secondary forest. The density of *F. mandshurica* (DBH ≥ 5cm) in secondary forests was 27 trees·ha^−1^, with young trees (5cm ≤ DBH< 10cm) predominating, accounting for 74%. In contrast, primary forests contained 12 large trees·ha^−1^. Regarding seedling recruitment, a survey of 200 plots (1m × 1m) revealed 100 seedlings in the secondary forest, 49% of which were<10cm in height, in the primary forest, there were only 125 seedlings, 59% of which were<10cm in height (**Figure 2**).

**Table 1:** Basic information of the two forests studied and characteristics of the environmental conditions of forest undercanopy during the growing seasons in 2021 and 2022.

Variable	Secondary forest	Primary forest
Species richness	25	18
Density (trees·ha^−1^)	1159	587
BA (m^2^·ha^−1^)	33.7	43.5
Soil pH	5.52	5.58
STC (mg·g^−1^)	56.8	80.4
STN (mg·g^−1^)	4.5	7.1
2021 PAR (μmol·m^−2^·s^−1^)	25.5	20.2
2021 T_air (°C)	14.2	13.9
2021 T_soil (°C)	12.2	13.0
2021 SM (%)	21.5	34.0
2022 PAR (μmol·m^−2^·s^−1^)	29.1	23.1
2022 T_air (°C)	13.9	13.7
2022 T_soil (°C)	12.9	12.5
2022 SM (%)	29.0	31.9

**Figure 3:**
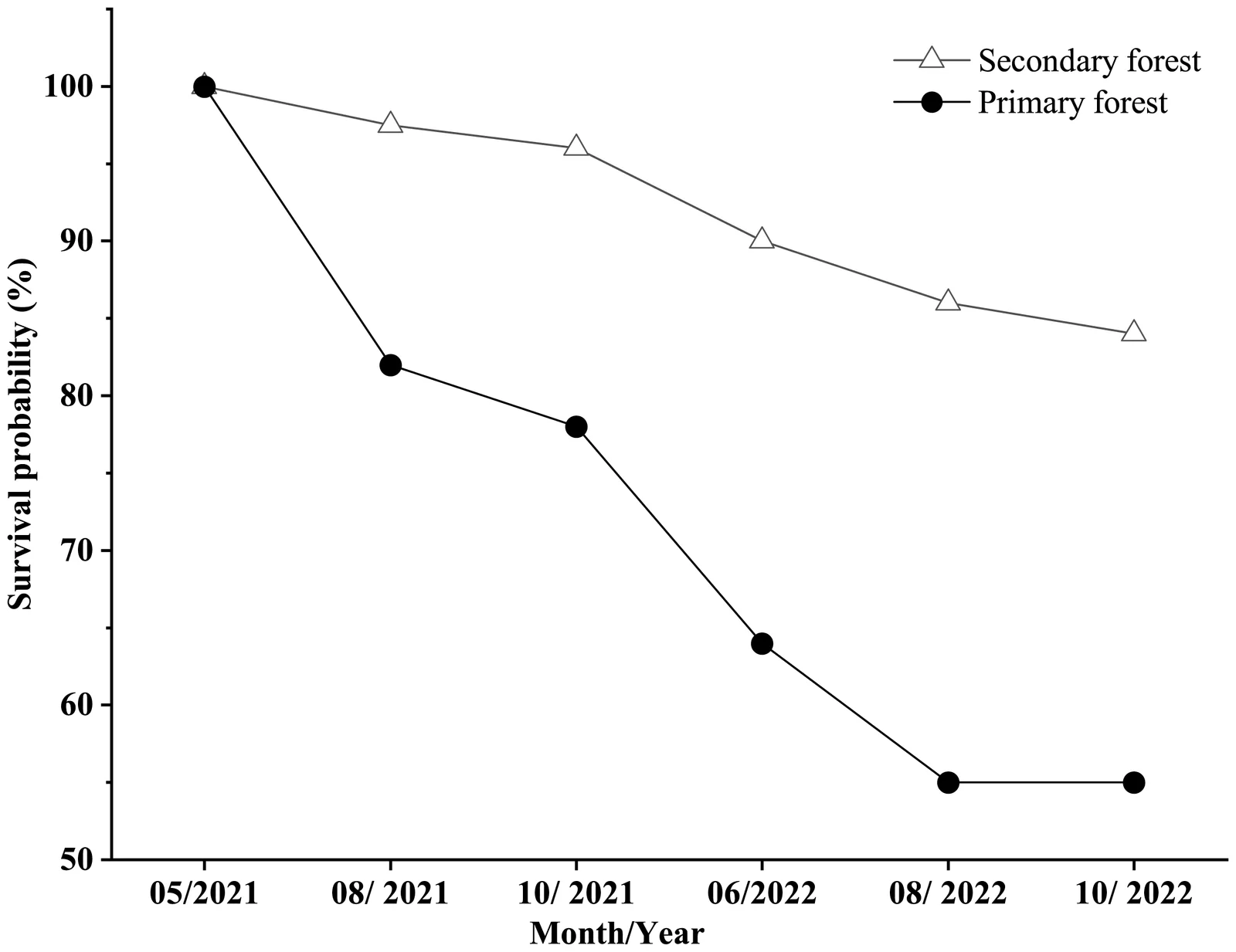
Seasonal variation in survival of *F. mandshurica* seedlings in primary and secondary forests.

The survival rate of *F. mandshurica* seedlings in the secondary forest was higher than that in the primary forest in both stands. Specifically, based on results from October 2022 when monitoring concluded, the survival rate of *F. mandshurica* seedlings in the secondary forest gradually decreased from 100% to 84% (with the largest drop of 6% occurring between October 2021 and June of the following year), while in the primary forest, the survival rate dropped from 100% to 55% (**Figure 3**).

### Growth parameters

**Figure 4:**
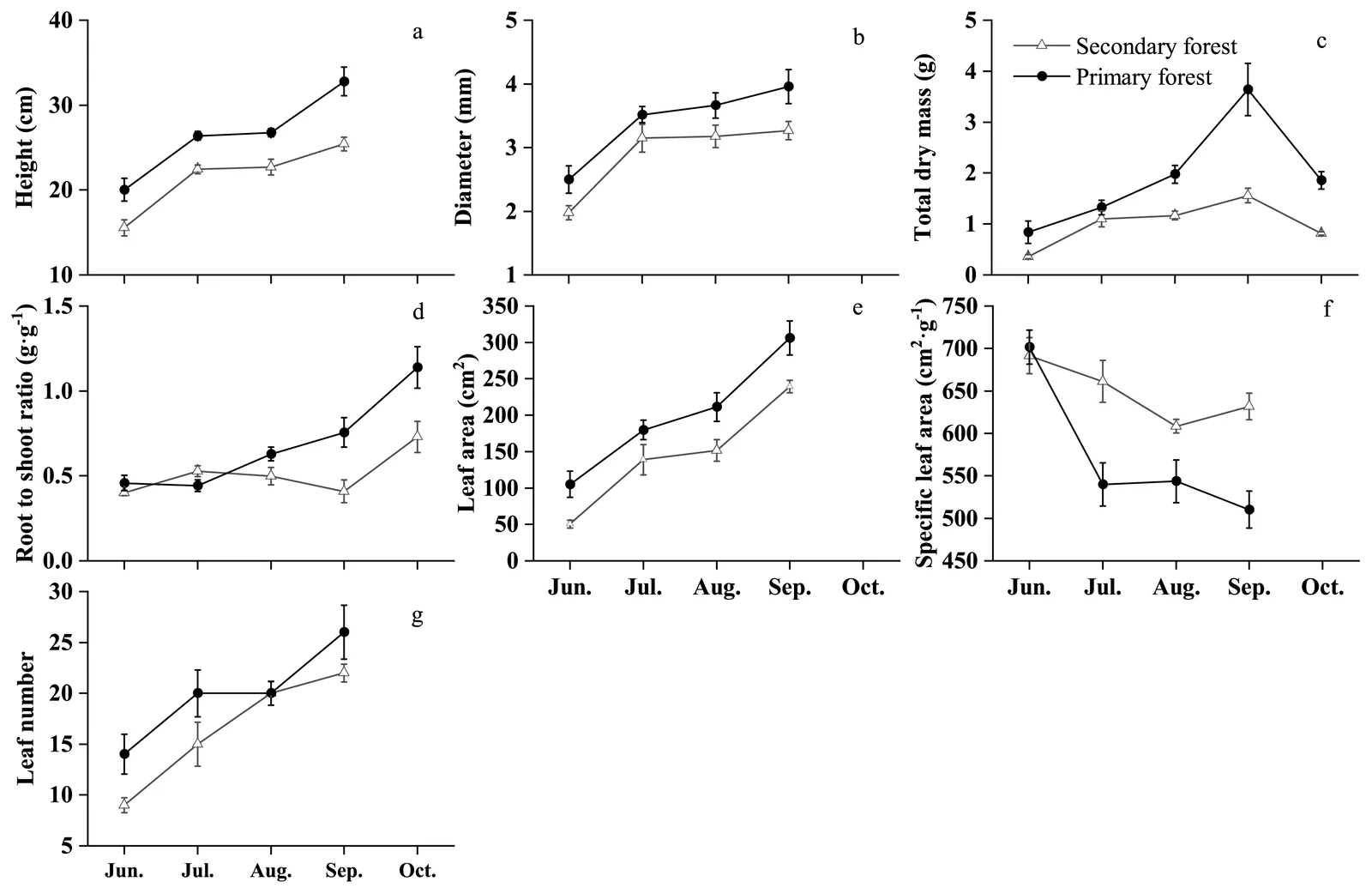
Seasonal dynamics of growth indicators of *F. mandshurica* seedlings in the primary and secondary forest.

In terms of seasonal dynamics, *F. mandshurica* seedlings exhibited two distinct growth pulses, occurring in June–July and August–September. Total dry mass increased considerably over the growing season, reaching 2.80g in September. The root-to-shoot ratio showed a gradual increasing trend over time. Leaf area increased steadily from June to September. Specific leaf area (SLA) declined markedly in June–July, after which the two stands diverged in their seasonal patterns. By the end of October, *F. mandshurica* seedlings in the secondary forest had increased in height by 9.87cm and in basal diameter by 1.29mm, whereas those in the primary forest had increased by 12.75cm and 1.46mm, respectively. Leaf area of primary forest seedlings was greater than that of secondary forest seedlings from June to September. In contrast, SLA of secondary forest seedlings was higher than that of primary forest seedlings from July to September. Leaf number of primary forest seedlings exceeded that of secondary forest seedlings in June–July and September (**Figure 4**).

### Seasonal biomass allocation

**Figure 5:**
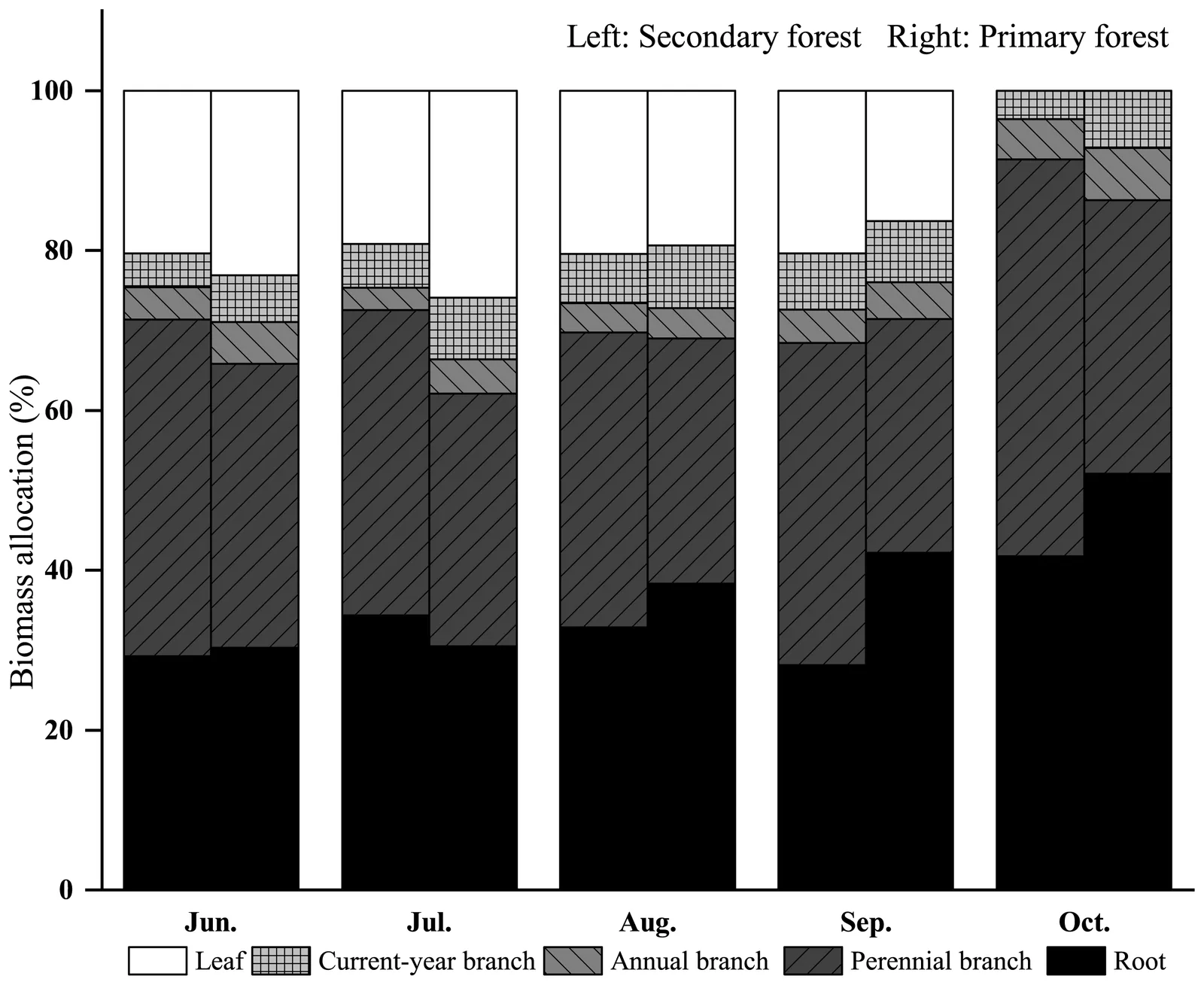
Seasonal dynamics of biomass allocation in *F mandshurica* seedlings under primary and secondary forest conditions.

The biomass allocation among organs of *F. mandshurica* seedlings differed significantly between forest types and exhibited distinct seasonal patterns. During the canopy closure period (June–September), root biomass allocation in the primary forest seedlings gradually increased, while it gradually decreased in the secondary forest (**Figure 5**). Regarding branch allocation, the biomass allocation of current-year branches in the secondary forests continued to increase and was higher than that in the primary forests, the allocation of one-year-old branches in both stands remained generally stable, for multi-year-old branches, the primary forests showed a trend of first decreasing and then stabilizing, while the secondary forest first decreased and then increased. Regarding leaf biomass allocation, the secondary forest remained generally stable, while the primary forest first increased and then decreased sharply. During the leafless period (October), root biomass allocation in the primary forests remained high, but the gap between the two forest types narrowed (**Figure 5**).

### Seasonal dynamics of NSC

**Figure 6:**
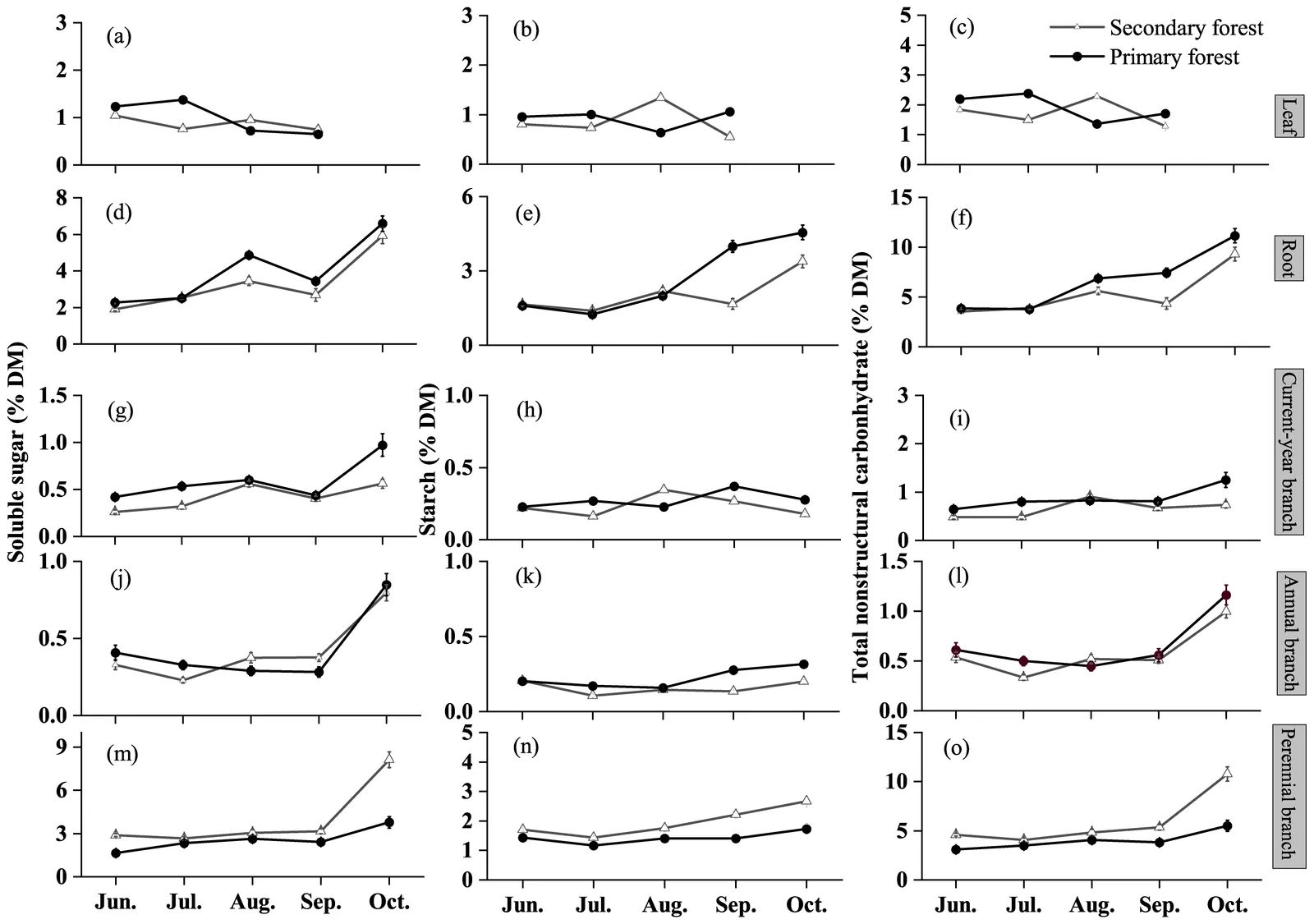
Seasonal dynamics of NSCs in different tissues of *F. mandshurica* seedlings in the primary and secondary forest. Panels **(a–c)**: Soluble sugar and Starch and total nonstructural carbohydrate concentration in leaf; **(d–f)**: Soluble sugar and Starch and total nonstructural carbohydrate concentration in root; **(g–i)**: Soluble sugar and Starch and total nonstructural carbohydrate concentration in current‑year branch; **(j–l)**: Soluble sugar and Starch and total nonstructural carbohydrate concentration in annual branch; **(m–o)**: Soluble sugar and Starch and total nonstructural carbohydrate concentration in perennial branch.

Leaf soluble sugar in both stands decreased and then stabilized during summer, whereas leaf starch in the secondary forest showed an increasing trend in August. Root soluble sugar and total NSC concentrations exhibited consistent trends across stands, with the former showing an initial increase followed by a decrease, and the latter showing a gradual increase. From August to October, root soluble sugar, starch, and total NSC concentrations were higher in the primary forest seedlings than in the secondary forest seedlings. Current-year branch NSC concentrations varied little, with no significant difference between stands in September, but soluble sugar and total NSC concentrations in the primary forest increased markedly in October. One-year-old branch NSC concentrations also showed little variation, but both stands exhibited increasing trends in October. From June to October, perennial branch soluble sugar, starch, and total NSC concentrations in the secondary forest were higher than those in the primary forest, with a notable increase in October. Overall, roots had the highest concentrations of soluble sugar, starch, and total NSC, while one-year-old branches had the lowest (**Figure 6**).

### Seasonal carbon gain in leaves

**Figure 7:**
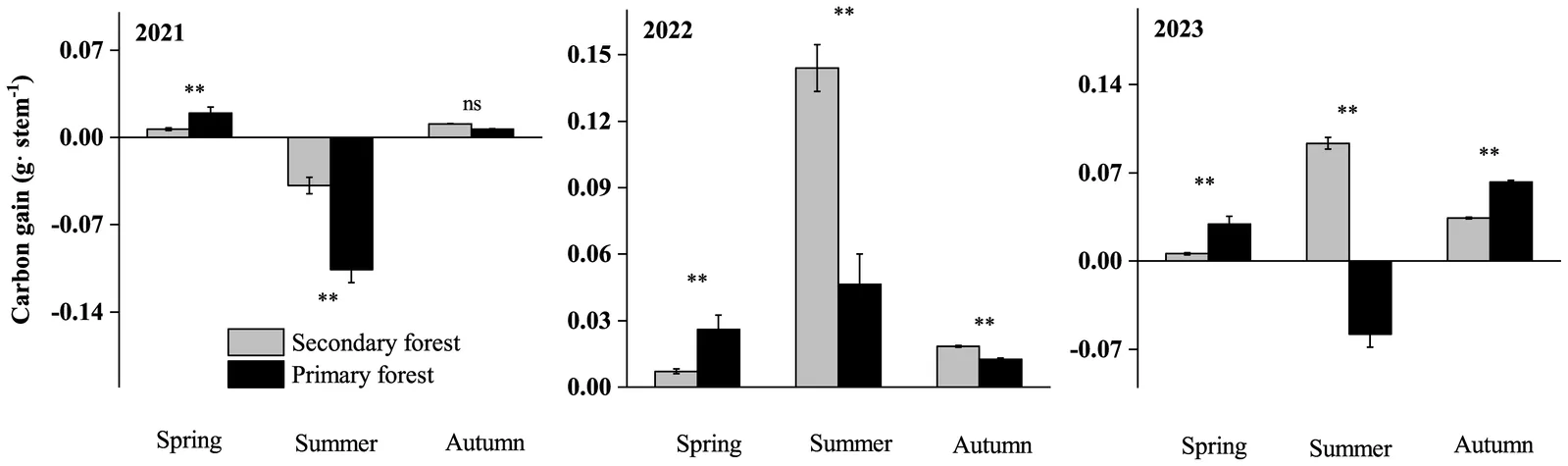
Simulated carbon assimilation of understory *F. mandshurica* seedlings across years and seasons. ** indicate extremely significant differences (*p*<0.01), and ns denotes non-significant differences.

Data from the three-year period showed that carbon assimilation in *F. mandshurica* seedlings was positive in both spring and autumn, indicating that they achieved positive carbon gains by utilizing differences in canopy phenology during early spring and late autumn. Summer carbon assimilation varied between the two forest stands across years, both were negative in 2021, both were positive in 2022, and in 2023, the secondary forest was positive while the primary forest was negative, which is related to differences in light availability over the three years (**Figure 7**). Accordingly, the annual net carbon gains for the secondary and primary forests were as follows, −0. 021g and −0. 080g in 2021, 0. 169g and 0. 085g in 2022, and 0. 133g and 0. 034g in 2023.

### Growth performance of seedlings during the growing season in the nursery

**Figure 8:**
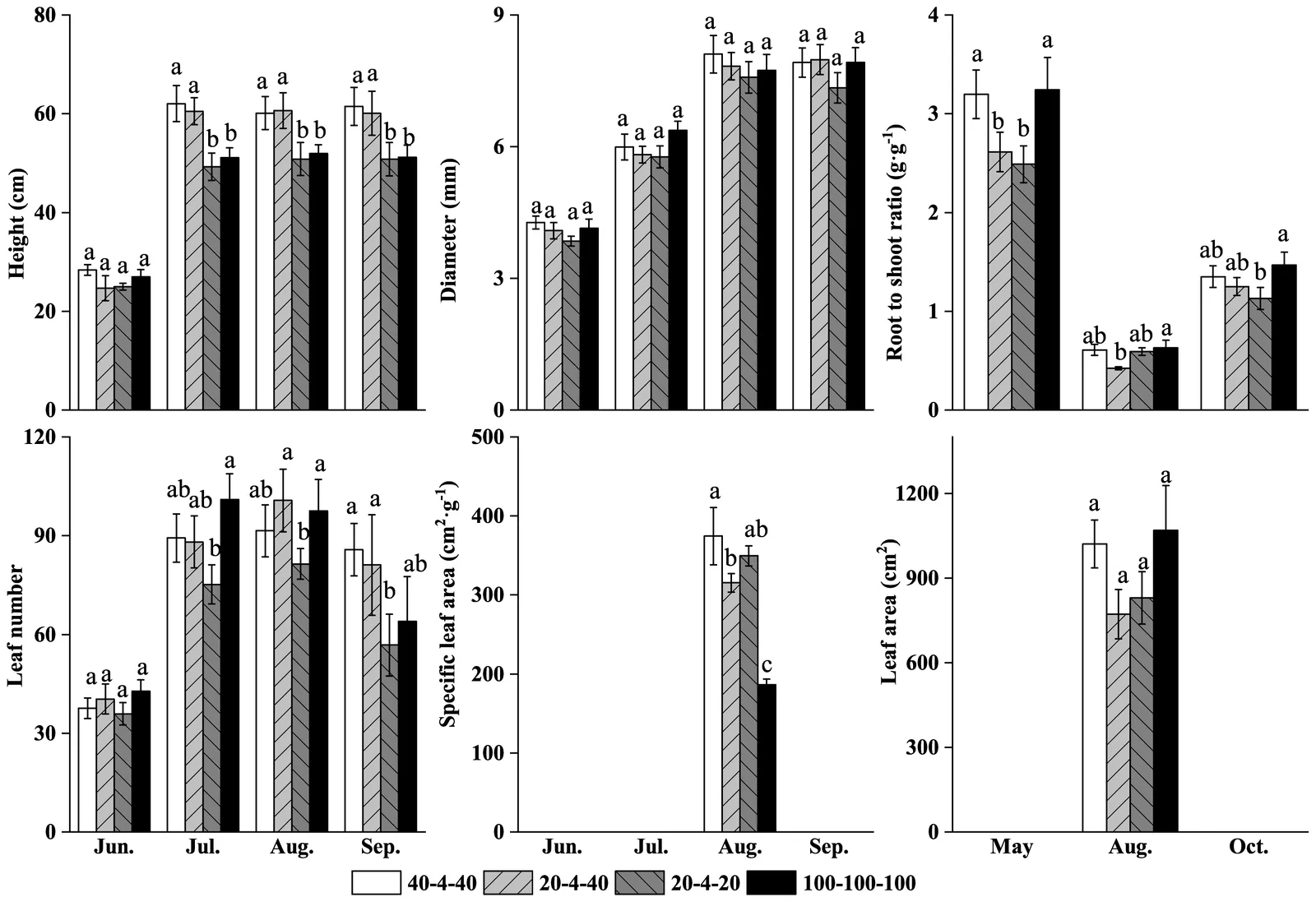
Seasonal variation in growth characteristics of *F. mandshurica* seedlings under different shading treatments. The three-digit codes represent seasonal light transmittance (spring–summer–autumn) under simulated canopy conditions: 40-4-40: Simulated secondary forest with 40%, 4% and 40% light transmittance in spring, summer and autumn, respectively. 20-4-40: Simulated primary forest with elevated autumn light (20% in spring, 4% in summer, 40% in autumn), 20-4-20: Simulated natural primary forest (20%, 4%, 20% transmittance for the three seasons). 100-100-100: Full light open control group with 100% transmittance across all three seasons. All data are expressed as mean ± standard error (SE, n = 3). Different lowercase letters indicate significant differences among various treatments in the same month. The same applies below.

There were significant differences (*p*<0. 05) in seedling height, root-to-shoot ratio, specific leaf area, and number of leaves among the different treatments except in June. Net growth in seedling height and stem diameter occurred in June–July and June–August, respectively, the annual net increment in seedling height under simulated primary forest was lower than that under simulated secondary forest and full-sunlight treatments. Specific leaf area was significantly highest under simulated secondary forest conditions and lowest under full sunlight, from August to September, the number of leaves was significantly higher under simulated secondary forest conditions than under simulated primary forest conditions (**Figure 8**).

**Figure 9:**
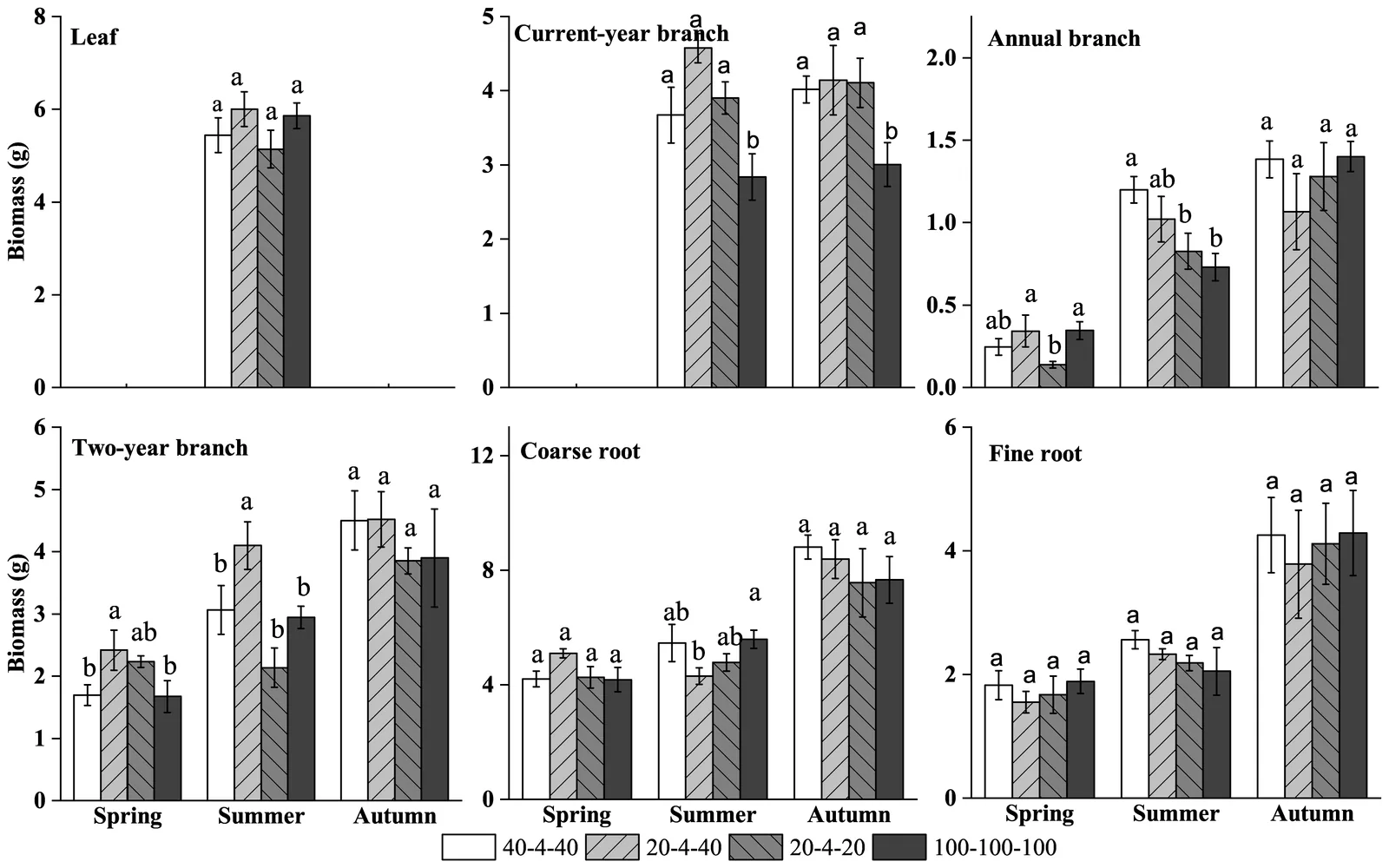
Seasonal variation in biomass of different tissues of *F. mandshurica* seedlings under different shading treatments.

With the exception of coarse and fine roots, biomass growth of the other organs occurred primarily from spring to summer. However, the biomass of coarse and fine roots increased significantly in autumn. The biomass of all organs except current-year branch under the simulated secondary forest treatment was higher than that under the simulated primary forest treatment in autumn (**Figure 9**).

**Figure 10:**
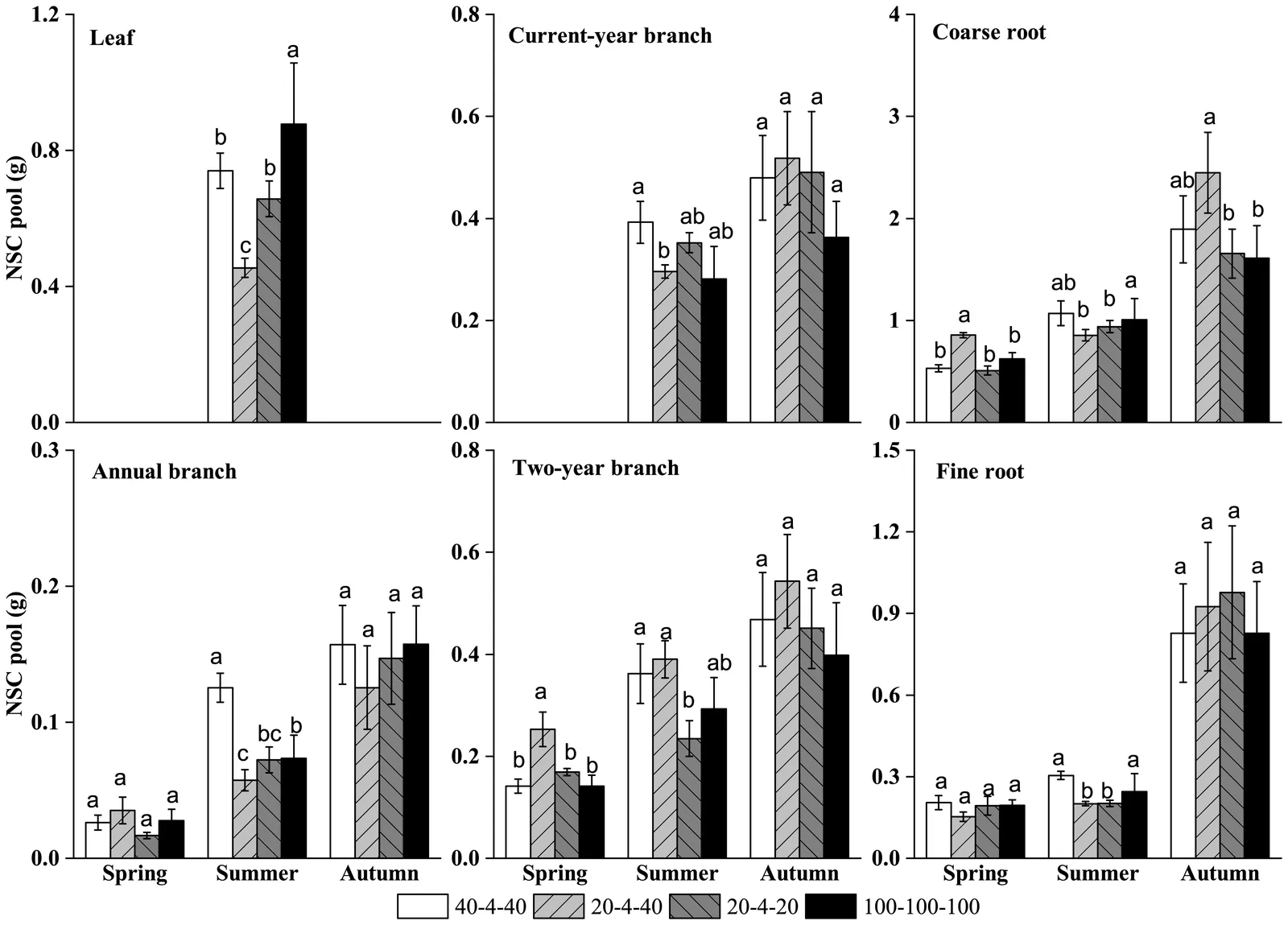
Seasonal variation in NSC pools of *F. mandshurica* seedlings in different tissues under different shading treatments.

With the exception of fine roots, NSC pools in all organs increased continuously from spring through summer to autumn. Under simulated primary forest conditions, NSC pools in coarse roots, fine roots, and first-year branches were all lower than those under simulated secondary forest conditions. In autumn, NSC pools in all organs increased significantly, serving as nutrient reserves for overwintering and leaf unfurling the following year. (**Figure 10**).

## Discussion

### Exploitation of the high-light gap is key to successful regeneration

Light availability fundamentally determines whether understory seedlings can successfully regenerate (Wang and Wang, 2023). This is especially true in temperate deciduous forests, where two distinct high-light gaps occur one in early spring before canopy leaf-out, and another in late autumn after leaf fall begins. These gaps arise from the phenological dynamics of overstory trees and provide critical opportunities for carbon acquisition by understory plants (Depauw et al., 2020; Dox et al., 2020; Gamhewa et al., 2025). Our light monitoring data confirmed this pattern, across both stand types, understory light levels remained below 20 μmol·m^−2^·s^−1^ during summer (approximately 2% of full sunlight), well below the threshold for sustaining long-term seedling survival. In contrast, light availability increased substantially during both spring and autumn gaps. Notably, despite the extremely low summer light, *F. mandshurica* seedlings in the secondary forest maintained positive carbon gain during this period, while autumn contributed even greater carbon accumulation than spring despite its shorter duration (**Figure 7**). This pattern aligns with the phenological escape framework proposed by Lee and Ibáñez (2021), who demonstrated that temperate tree seedlings rely on high-light gaps before canopy closure in spring and after leaf fall in autumn to acquire a substantial portion of their annual carbon budget. Our findings confirm phenology as a key determinant of seedling regeneration, yet the mechanisms linking phenological shifts to seedling fitness remain underexplored, underscoring the value of integrating multi-trait functional ecology with seasonal phenology to advance understanding of understory regeneration. These findings confirm that the seasonal light regime, particularly the occurrence and duration of high-light gaps, plays a decisive role in seedling carbon balance and regeneration success.

In temperate deciduous forests, understory seedlings often adopt a phenological escape strategy to exploit high-light gaps and achieve early carbon accumulation, and *F. mandshurica* seedlings are no exception (Lee et al., 2024). In secondary forests, the seedling growing season spans May 12 to September 17, while the canopy closure period lasts from May 25 to September 5. This means that seedlings in secondary forests can access approximately two weeks of high-light conditions in both spring and autumn, which constitutes the core phase of their annual carbon accumulation. In contrast, seedlings in primary forests are significantly suppressed due to the longer canopy closure period, resulting in negative annual net carbon gains in some years. What are the consequences of these phenological differences? In secondary forests, *F. mandshurica* seedlings can successfully develop into saplings (with a density of up to 20 individuals per hectare at a breast height diameter of 5–10 cm), seedlings in secondary forests can successfully develop into saplings, whereas those in primary forests fail to do so. The high-light gap in secondary forests lasts about 5 weeks, whereas in primary forests it is shortened by nearly 40%, leading to insufficient carbon accumulation in seedlings and resulting in carbon starvation. This finding is consistent with Valladares et al. (2016), who reported that early carbon deficits are the primary driver of seedling mortality.

In response to seasonal changes in light availability, *F. mandshurica* seedlings exhibit significant plasticity in their carbon accumulation strategies. Three-year data show that carbon assimilation in both spring and autumn is positive, allowing seedlings to effectively utilize phenological differences to achieve positive carbon gains. However, summer carbon assimilation and annual net carbon gains exhibit interannual fluctuations (**Figure 7**). This study supports previous findings that deciduous woody plants acquire most of their annual carbon during the leafless period, but differs from reports emphasizing the dominant role of spring phenological escape by demonstrating that autumn phenology also exerts a decisive influence on the carbon balance of *F. mandshurica* seedlings (Lee and Ibáñez, 2021). Carbon assimilation early in the growing season helps seedlings establish carbon reserves before canopy closure, enhancing their survival capacity during summer shading (Zhou et al., 2020; Lu and Keenan, 2022; Liu et al., 2023). In autumn, although aboveground growth tends to stagnate, seedlings can still accumulate carbohydrates through continued photosynthesis, ensuring survival through winter and supporting leaf expansion the following spring (Piper et al., 2022; Durand et al., 2024). Consistent with Givnish’s (1988) theoretical framework, plants optimize their carbon capture strategies under low-light conditions. However, unlike the findings of Valladares et al. (2016), who reported that annual carbon gains remained negative for most tree species under sustained low light, secondary forest *F. mandshurica* seedlings in this study achieved positive carbon gains in some years, whereas primary forest seedlings showed negative gains. This contrast highlights the critical role of high-light gaps especially the autumn gap in enabling seedlings to maintain a positive carbon balance even in partially shaded environments. This aligns with the view of Myers and Kitajima (2007) that carbon gain dynamics determine the shade tolerance of seedlings. Secondary forest seedlings, which maintained positive carbon gains and experienced smaller losses during carbon deficit years, demonstrate a greater capacity for carbon balance regulation, whereas primary forest seedlings, with negative annual carbon gains, fail to survive under sustained low light. Collectively, light was the dominant factor driving survival differences among treatments, whereas biotic factors (interspecific competition, neighbor interference) and abiotic factors (soil moisture, allelopathy, soil nutrients) may jointly regulated seedling performance. In summary, the higher light availability in secondary forests during spring and autumn provides a critical gap for carbon accumulation in *F. mandshurica* seedlings, enabling them to maintain a positive carbon balance during the summer canopy closure period (Liu et al., 2020b; Fernández-Milmanda and Ballaré, 2021). Which may be the most direct reason for their successful regeneration in secondary forests and their failure in primary forests.

### Aboveground allocation strategies are critical for seedling survival

The allocation of biomass among leaves, branches, and roots in seedlings varies with light availability, reflecting their adaptive strategies to different light environments (Balandier et al., 2022). During the summer canopy closure period (June to August), we observed contrasting patterns of biomass allocation between the two forest types. This study found that the above-ground biomass allocation of *F. mandshurica* seedlings in secondary poplar-birch forests showed a gradual seasonal increase, whereas seedlings in primary broad-leaved Korean pine forests exhibited the opposite trend with below-ground biomass allocation continuing to rise (**Figure 5**). This striking contrast reveals that *F. mandshurica* seedlings in the two forest types adopt distinctly different survival strategies, suggesting that above-ground biomass allocation plays a critical role in seedling survival under shaded conditions.

To adapt to changes in light availability, plants typically adjust functional traits such as seedling height and specific leaf area (Zhang et al., 2020). This study found that seedling height and leaf area were both higher in primary forests than in secondary forests, yet the specific leaf area was higher in secondary forest seedlings reflecting different survival logics (**Figure 4**). Seedlings in primary forests attempt to capture more light energy by increasing plant height and leaf area. Unfortunately, under extremely low-light conditions, the marginal benefit of this approach is very limited. In contrast, secondary forest seedlings opt to enhance light capture efficiency by increasing specific leaf area, a proactive optimization strategy that allows them to maintain a positive carbon balance even during the canopy closure phase. Research by Kong et al. (2023) also supports this finding, specific leaf area reaches its maximum at 15% relative light intensity, indicating that moderate shading does indeed promote the accumulation of photosynthetic products in leaves. This indicates that *F. mandshurica* possesses considerable phenotypic plasticity in leaf morphology which may also be one of the key physiological foundations for its successful regeneration in secondary forests.

The effectiveness of this above-ground allocation strategy varies between forest types. It promotes seedling survival in secondary forests, where high-light gaps are longer, but contributes little to survival in primary forests, where canopy closure is more prolonged. In secondary forests, seedlings allocate more resources to leaves and branches at the expense of the root system, with aboveground allocation peaking by September. This strategy works because secondary forests have extended periods of high light availability in spring and autumn during which seedlings maximize light capture efficiency to rapidly accumulate photosynthetic products and store carbon reserves for the summer canopy closure period. The situation in primary forests is entirely different, seedlings instead increase root investment and reduce leaf allocation (Herrera‐Ramírez et al., 2020). However, because the canopy closure in primary forests is higher and the closed-canopy period is longer, the marginal returns of above-ground photosynthesis are very limited (Luo et al., 2022). Increasing leaf investment yields little return, while increasing root investment fails to address the core issue of insufficient carbon uptake (Wang and Wang, 2023). This suggests that the effectiveness of above-ground allocation depends not only on the proportion but also on the timing secondary forest seedlings actively allocate resources to the aboveground parts when light is abundant (a carbon accumulation strategy), whereas primary forest seedlings passively increase above-ground allocation when light is limited (a carbon consumption strategy). Therefore, above-ground allocation promotes survival under shading conditions when high-light gaps are available, but it struggles to compensate for the carbon deficit when such gaps are absent (Dox et al., 2020; Montague et al., 2022).

### Enhanced autumn light can increase NSC reserves and survival rates

This study showed that *F. mandshurica* possesses a certain degree of shade tolerance, however, within the study area, its seedlings successfully regenerated in secondary poplar-birch forests but struggled to regenerate in primary broad-leaved Korean pine forests. Monitoring of PAR under the canopy revealed that while summer light conditions differed little between the two stands (**Figure 1**). There were significant differences in light availability during spring and autumn. These phenologically driven light differences played a key role in seedling regeneration, which was further validated in nursery experiments. Autumn light availability significantly affects the NSC dynamics of *F. mandshurica* seedlings by promoting carbon accumulation and storage before winter dormancy. Our study found that increasing autumn light under the canopy significantly increases NSC reserves in *F. mandshurica* seedlings, thereby improving survival rates. Whereas under simulated primary forest light conditions, seedlings experienced limited NSC accumulation due to insufficient autumn light, resulting in the highest summer mortality rates (**Figure 10**). Higher light levels in late autumn facilitate carbohydrate accumulation in seedlings, promoting growth and survival (Balandier et al., 2022). Conversely, prolonged shading during the mid-growing season may lead to insufficient carbon accumulation, ultimately triggering carbon starvation (Fernández-Milmanda and Ballaré, 2021; Fan et al., 2024). It is highly consistent with the view of Myers and Kitajima (2007) that NSC reserves are key to seedling survival under shaded conditions. In this study, NSC reserves in *F. mandshurica* seedlings were significantly higher under simulated secondary forest and autumn full-light conditions, providing ample nutrient reserves for overwintering and leaf unfurling the following year (Sarah et al., 2015). However, unlike Kobe’s (1997) conclusion that shade-tolerant tree species primarily rely on spring carbon reserves to overwinter. Our study found that autumn light plays an equally indispensable role in NSC accumulation in *F. mandshurica* seedlings, and in some years even contributes more than spring light. This finding is closer to that of Fridley (2012) deciduous plants acquire the majority of their annual carbon gain during the autumn leafless period.

It is estimated that 50%–80% of PAR in forest ecosystems reaches the understory in the form of light patches, which are crucial for seedling carbon accumulation and survival (Pritzkow et al., 2020). In this controlled experiment, the intensity and duration of light patches in the primary forest were insufficient to maintain normal carbon balance in *F. mandshurica* seedlings. Whereas the secondary forest, with longer light patch duration and higher light intensity, enabled more seedlings to successfully regenerate. Nursery experiments further confirmed that enhanced autumn light exposure helps improve seedling survival rates under primary forest conditions. Based on these findings, we propose a seasonal light influence model, in spring and autumn, source activity exceeds sink activity, resulting in increased NSC reserves and positive carbon gains, during the summer canopy closure period, the opposite occurs. This model is consistent with the observational results of Hoch et al. (2003) on the seasonal dynamics of NSC in temperate deciduous trees, namely that carbon reserves peak at the end of the growing season and are significantly depleted during dormancy. Future research could further investigate the juvenile stage of water-birch in secondary poplar-birch forests to refine the understanding of its full life-history regeneration mechanisms (Peng et al., 2024).

## Conclusion

In summary, early spring (pre-canopy closure) and late autumn (post-leaf fall) gap greatly improved understory light, raising net carbon gain and NSC reserves in *F. mandshurica* seedling leaves. This highlights the decisive role of light availability during these phenological gaps and the non-growing season in regulating seedling survival and growth of *F. mandshurica*. Specially, in spring, seedlings achieve efficient carbon fixation by taking advantage of ephemeral high light. During the shaded summer, seedlings rely on stored NSC to sustain basal metabolism and survival, and enhance light capture by allocating more biomass aboveground and increasing specific leaf area to endure low-light stress. With recovered light availability in autumn, seedlings resume carbon assimilation and preferentially allocate photosynthates to roots and perennial branches for NSC storage, supporting winter survival and leaf flush in the following spring. Thus, light conditions in early spring and late autumn directly determine the annual carbon balance of *F. mandshurica* seedlings.

## Data Availability

The original contributions presented in the study are included in the article/supplementary material. Further inquiries can be directed to the corresponding author.
